# Exploring Types of Information Sources Used When Choosing Doctors: Observational Study in an Online Health Care Community

**DOI:** 10.2196/20910

**Published:** 2020-09-16

**Authors:** Shuang Zhang, Jying-Nan Wang, Ya-Ling Chiu, Yuan-Teng Hsu

**Affiliations:** 1 College of International Finance and Trade Zhejiang Yuexiu University of Foreign Languages Zhejiang China; 2 Research Institute for Modern Economics and Management Zhejiang Yuexiu University of Foreign Languages Zhejiang China; 3 College of International Business Zhejiang Yuexiu University of Foreign Languages Zhejiang China; 4 Research Center of Finance Shanghai Business School Shanghai China

**Keywords:** information source, decision making, online reviews, online health care community, doctor, health information

## Abstract

**Background:**

Patients attempt to make appropriate decisions based on their own knowledge when choosing a doctor. In this process, the first question usually faced is that of how to obtain useful and relevant information. This study investigated the types of information sources that are used widely by patients in choosing a doctor and identified ways in which the preferred sources differ in various situations.

**Objective:**

This study aims to address the following questions: (1) What is the proportion in which each of the various information sources is used? (2) How does the information source preferred by patients in choosing a doctor change when there is a difference in the difficulty of medical decision making, in the level of the hospital, or in a rural versus urban situation? (3) How do information sources used by patients differ when they choose doctors with different specialties?

**Methods:**

This study overcomes a major limitation in the use of the survey technique by employing data from the Good Doctor website, which is now China's leading online health care community, data which are objective and can be obtained relatively easily and frequently. Multinomial logistic regression models were applied to examine whether the proportion of use of these information sources changes in different situations. We then used visual analysis to explore the question of which type of information source patients prefer to use when they seek medical assistance from doctors with different specialties.

**Results:**

The 3 main information sources were online reviews (OR), family and friend recommendations (FR), and doctor recommendations (DR), with proportions of use of 32.93% (559,345/1,698,666), 23.68% (402,322/1,698,666), and 17.48% (296,912/1,698,666), respectively. Difficulty in medical decision making, the hospital level, and rural-urban differences were significantly associated with patients’ preferred information sources for choosing doctors. Further, the sources of information that patients prefer to use were found to vary when they looked for doctors with different medical specialties.

**Conclusions:**

Patients are less likely to use online reviews when medical decisions are more difficult or when the provider is not a tertiary hospital, the former situation leading to a greater use of online reviews and the latter to a greater use of family and friend recommendations. In addition, patients in large cities are more likely to use information from online reviews than family and friend recommendations. Among different medical specialties, for those in which personal privacy is a concern, online reviews are the most common source. For those related to children, patients are more likely to refer to family and friend recommendations, and for those related to surgery, they value doctor recommendations more highly. Our results can not only contribute to aiding government efforts to further promote the dissemination of health care information but may also help health care industry managers develop better marketing strategies.

## Introduction

### Background

Promoting patient choice can encourage competition among health care providers, which is likely to make health care more responsive to patient needs, enhance equity in care, and improve efficiency or quality as a result of effects such as reductions in wait times and costs [1-3]. Some studies have pointed out that consumer-directed health care does not always control costs better than other systems and that it has no significant effect on quality improvement [4,5]. The focus of this study is not on exploring the question of whether consumer-directed health care policies should be implemented but rather on understanding more fully the types of information sources consumers use when choosing a doctor. When they are able to make a rational choice, patients themselves can find a quality provider by weighing information from different sources. In practice, however, it is difficult for most patients to make fully rational choices [6-10], a difficulty which may result from certain individual characteristics. One of the most important characteristics is the patient's health literacy (ie, the capacity to access, process, and understand basic health information) [8,11-13]. Whether patients intend to actively use health care information when they choose between providers is another relevant question [14-16]. Furthermore, if patients encounter barriers to accessing information, such as short times for making decisions, geographical barriers [15], distrust of information [17], and information overload [18,19], this can also lead to bias in the decision-making process. Therefore, understanding which types of information sources are used widely for choosing doctors and how patients' preferences for information sources change under various circumstances will be useful for mitigating the barriers to information dissemination and for improving the decision-making process for patients.

The information sources that patients can use when choosing a doctor or health care provider are diverse. The mainstream literature has explored how the patient's choice is affected by public reports that compare the quality of health care providers. Although patients generally agree that such comparative reports are important, relatively few can understand or use them [15,19-21]. In order to take into account the widest possible variety of information sources for choosing doctors, this study divided information sources into 6 categories: online reviews (OR), family and friend recommendations (FR), doctor recommendations (DR), random registration (RR), multiple reasons (MR), and others (OTS). The first 3 types of information sources require further explanation.

First, with the growth of mature online health care communities (OHC) [22-26], many people can use online reviews in the OHC to share their opinions on the quality of their doctors. The function of the online health care community is now more than merely providing people with opportunities to share their ideas on the experience of seeing a doctor. The OHC now provides nontraditional channels and approaches to health care services, such as the use of electronic communication to query or improve a patient’s clinical condition [27,28]. Since this type of information is usually free to the public, patients can refer to it easily when choosing a doctor. However, some potential complications make online reviews less credible. For example, doctors with lower qualifications are less likely to be rated online, and doctors' online scores are often exaggerated at the upper end of the quality spectrum [29]. In addition, avoiding shilling attacks remains a major challenge for all online review platforms [30]. Thus, relying entirely on online reviews for choosing a doctor is not an optimal solution, and the patient's decision-making process sometimes requires additional reference to other, more reliable information sources.

Second, while online reviews provided by mere acquaintances or by individuals who do not know the patient at all can be considered weak-tie recommendation sources, recommendations from friends or family numbers are strong-tie sources [31]. The main advantage of strong-tie sources is that they allow for an evaluation of alternatives based on the individual’s situation. Specifically, compared with online reviews, family and friend recommendations are more likely to be a good source of information regarding affective cues rather than instrumental cues [32]. Another advantage is that patients usually do not have to worry about the deliberate falsification of information from friend recommendations. Nevertheless, family and friend recommendations are usually based on personal experience rather than on professional advice, which is based on professional medical knowledge. If a patient needs professional advice to choose the right doctor, the most common practice is to refer to a specialist, chosen with the assistance of a general physician. In addition, with the development of information communication technology, online medical consultations are becoming more and more popular [27,33], allowing doctors to answer patients' questions online. It should be noted that since the doctor-patient relationship is one of the most complex among all interpersonal relationships [34], it is not straightforward to determine whether doctor recommendations should be categorized as strong-tie or weak-tie sources.

Taken together, since these 3 types of information sources (ie, online reviews, family and friend recommendations, and doctor recommendations) do not have the same characteristics, understanding patients’ preference to use one or another of them in different situations can be useful for facilitating the transfer of information in the health care system.

Finally, we briefly introduce the 3 information sources that have not been fully explained. First, random registration means that patients do not deliberately choose a doctor by referring to any particular information. Second, the multiple reasons designation refers to a situation in which a patient uses multiple information sources for choosing a doctor; for example, patients may look up doctor reviews online and then ask friends which doctor is better. Third, we refer to situations that cannot be categorized in terms of the first 5 information sources as others, a step which ensures that our classification criteria cover all possibilities.

### The Research Problem

Understanding how patients choose doctors in different situations is important. However, due to considerations of cost, research conducted in the form of traditional questionnaires is often limited in terms of sample size, regional representativeness, and the diversity of specialties represented in the sample. This study overcomes this difficulty by collecting a large amount of data from an online health care community. Specifically, we address 3 main research questions. First, while some studies have discussed the impact of online physician evaluations or hospital evaluations on patient decision making [8,11,35,36], there are no studies, to the best of our knowledge, that specifically address the proportions in which different information sources are used in selecting doctors. Thus, our first research objective is to determine the proportions of use of the various information sources used to select physicians. This will give us a fuller picture of the current state of public access to medical information.

Second, there are a number of factors that can affect the decision-making process of patients seeking medical care. From a decision-making difficulty perspective [32], if surgical treatment is involved, patients need to give more careful consideration to choosing a doctor. From the perspective of the patient's external environment, urban-rural differences may contribute to differences in information accessibility or correspond to differences in the patient's medical knowledge [28]. From the point of view of the reputation of medical institutions, an official announcement of the hospital ratings can influence the perception of reliability that patients have toward doctors [11,35]. Thus, the second research objective is to explore how the information source used in the patient's choice of doctor is affected by the treatment method, the size of the city where the hospital is located, and the hospital level.

Third, regarding most medical management or decision-making issues, the impact of the particular medical specialty area is clear. For example, compared with other specialties, physicians in the gynecology/obstetrics and pediatrics specialty areas are more willing to contribute to online health care communities [26]. This study employed a large amount of data collected from the online medical community, so the sample included data related to a wide variety of specialties. This facilitated our exploration of the third research question: how does the information source that patients use change when patients choose a doctor with a different specialty?

## Methods

### Data Collection and Processing

We collected a large amount of public data from the Good Doctor website to explore our research questions. Founded in 2006, the Good Doctor website is now China's leading OHC. As of December 2019, this website contains the online review profiles of 610,000 doctors. Of these, about 220,000 doctors who have been certified by this website are able to create their own pages and interact directly with patients online. Moreover, we chose this OHC for two reasons. First, the Good Doctor website has provided standard options for replying to the question “Why did you choose this doctor?” since October 2016, and this allowed us to directly observe the information source used in the patient's choice of doctor. Second, since it is one of the most popular OHCs in China, many recent studies have used data sources from this website for exploring various research questions [24,26,33,34], which implies that the data collected from the site are reliable and representative. Our data collection procedure is described in detail below.

**Figure 1:**
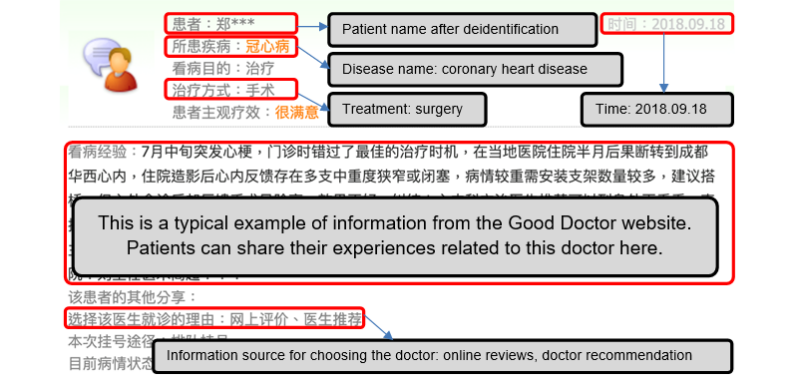
Screenshot of a review on the Good Doctor website.

**Figure 2:**
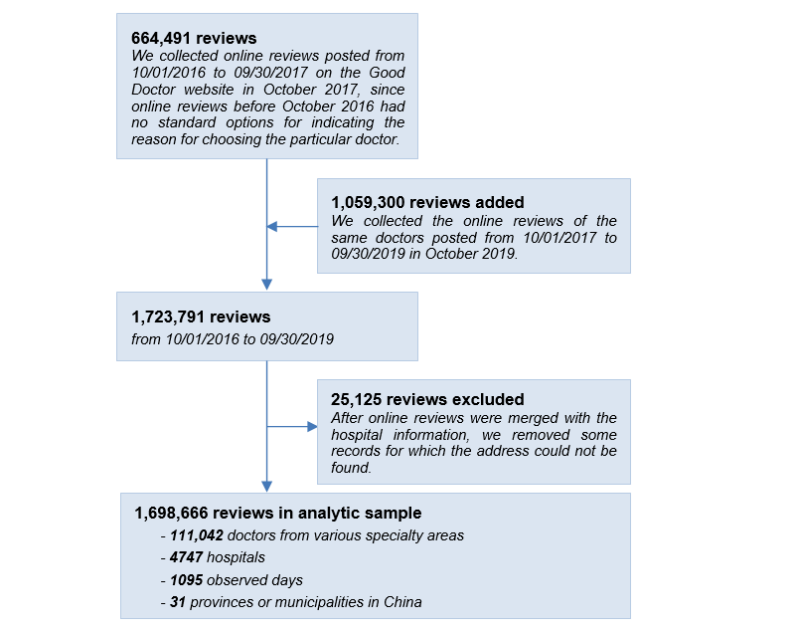
The sample collection process and characteristics.

As with most OHCs, patients can provide online reviews on the Good Doctor website to share their experiences of seeing doctors. Figure 1 represents a typical example of a review; each review contains textual content, the patient name after deidentification, a time stamp, the name of the disease, the treatment received, and the information source used for choosing that particular doctor. As the time stamps of online reviews are removed by the system after 2 years, we collected data in 2 phases for the purpose of making use of a longer sample period. First, in October 2017, we started to collect all reviews posted on the Good Doctor website from October 1, 2016, to September 30, 2017. In this phase, 664,491 reviews were added to our sample. Two years later, we repeated the same data collection procedure and obtained 1,059,300 reviews posted from October 1, 2017, to September 30, 2019. In addition to these reviews, we also collected data about the hospitals and departments to which the doctors belonged. The address and the level of each hospital can be obtained from other pages of the Good Doctor website. After online reviews were merged with the hospital information in our sample, 25,125 reviews were excluded due to the corresponding address of the hospital being missing in the data. Finally, we retained 1,698,666 reviews in our sample, including reviews with information on 111,042 doctors from various specialty areas, 4747 different hospitals, 1095 observed days, and 31 provinces or municipalities in China. The data collection process is displayed in Figure 2. It is important to note that since all patients have been deidentified in the data and only aggregated results are reported in this manuscript, this work meets the ethical requirements for academic research.

### Measures

In light of the kinds of data collected from the Good Doctor website, several variables involved in this study can be defined formally. First, when patients are filling out reviews, the Good Doctor website provides 4 defined options that can be indicated as the information source used to choose the doctor: online reviews, family and friend recommendations, doctor recommendations, and random registration. In addition to these 4 options, the system allows patients to write any other information source, and patients can select more than one type of information source. For convenience, we denote the former option as others and the latter as multiple reasons. With this setup, we were able to ensure that each item in the sample was clearly categorized in terms of using a particular information source. It is also worth noting that we looked carefully at the actual content written in the OTS option, and we found most of the indicated sources were very similar in nature to the RR option. This study focuses on discussing the 3 most common information sources, OR, FR, and DR. The related results from reviews that indicate the other sources are included for the reader's reference only.

In order to investigate factors that may affect patients' decisions about using the information sources to choose doctors, this study considers 3 other variables that can also be observed from the website. First, the patient can indicate the way their condition was treated, such as medication, counseling, surgery, or other means, in their review. Because surgical treatment is usually a decision involving greater deliberation for members of the general public, a patient’s consideration of whether to undergo surgery may result in a tendency to use certain information sources in choosing a doctor. Hence, we defined a dummy variable for treatment. When the treatment involved surgery, treatment=1; otherwise, treatment=0. Table 1 shows that up to 45.15% (766,933/1,698,666) of the patients in our sample were treated in a surgery-related manner. Second, we defined a dummy variable to indicate the hospital level, set to 1 if the doctor who was reviewed by the patient was from a tertiary hospital and set to 0 otherwise. Table 1 indicates that approximately 92.73% (1,575,216/1,698,666) of patients chose doctors from hospitals in the tertiary category, which is the official certification for the highest-quality hospitals. Third, because we want to understand how the degree of regional development affects the patients’ use of information sources in choosing doctors, we further defined a dummy variable for city, denoting the level of the city. Specifically, city was set to 1 if the doctor was from Beijing, Shanghai, Shenzhen, or Guangzhou, and otherwise it was set to 0. Table 1 shows that 45.35% (770,384/1,698,666) of the doctors in our sample were in first-tier cities in China.

**Table 1:** Descriptive statistics of variables.

Variable		Observation, n	Proportion, % (95% CI)
**Information source for choosing the doctor**			
	Online Reviews (OR)	559,345	32.93 (32.86-33.00)
	Family and friend recommendations (FR)	402,322	23.68 (23.62-23.75)
	Doctor recommendations (DR)	296,912	17.48 (17.42-17.54)
	Multiple reasons (MR)	131,648	7.75 (7.71-7.79)
	Random registration (RR)	111,800	6.58 (6.54-6.62)
	Others (OTS)	196,639	11.58 (11.53-11.62)
**Does the treatment include surgery? (TREATMENT)**			
	Yes (1)	766,933	45.15 (45.07-45.22)
	No (0)	931,733	54.85 (54.78-54.93)
**Is this doctor from a tertiary hospital? (HOSPITAL)**			
	Yes (1)	1,575,216	92.73 (92.69-92.77)
	No (0)	123,450	7.27 (7.23-7.31)
**Is this doctor from a big city? (CITY)**			
	Yes (1)	770,384	45.35 (45.28-45.43)
	No (0)	928,282	54.65 (54.57-54.72)
Total		1,698,666	100.00

### Statistical Analysis

To investigate the research question about which factors affect the selection of an information source when choosing a doctor, we looked at the impact of the above 3 dummy variables (ie, treatment, hospital, and city) on the choice of the information source. We used data visualization techniques to facilitate an understanding of the main findings of this research. Moreover, we formally adopted multinomial logistic regression to examine whether these findings were statistically significant. Multinomial logistic regression is an extension of binary logistic regression that allows the nominal dependent variable to belong to more than two categories and uses maximum likelihood estimation to evaluate the log odds of the outcomes. In practice, to deal with a multinomial logistic regression model with *K* possible outcomes, it is usual to estimate *K-1* independent binary logistic regression models, in which one outcome is chosen as a baseline and then the other *K-1* outcomes are compared to this baseline.

In this study, we consider 3 models with different baselines. To simplify the expression model, we denote *IS_1_, IS_2_, … , IS_6_* as OR, FR, DR, MR, RR, and OTS, respectively. Then, given OR (one outcome of the dependent variable) as the baseline, we construct model 1 with the following form:

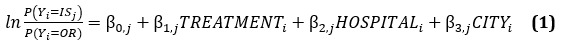



In the above equation*,* *j*=2, 3, 4, 5, or 6. Note that this model introduces 5 separate sets of regression coefficients for each possible outcome. For example, *j*=2 means the regression coefficients for the outcome are for FR. Hence, β_1,2_, β_2,2_, and β_3,2_ represent how the log odds ratio of FR versus OR will change if 3 independent variables (ie, TREATMENT, HOSPITAL, and CITY) move from 0 to 1, respectively. Similar to model 1, we also define model 2 as having the following form:

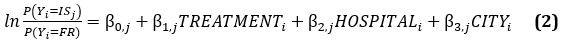



In the above equation*,* *j*=1, 3, 4, 5, or 6. Similarly, we define model 3 as having the following form:

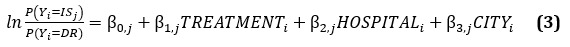



In model 3, *j*=1, 2, 4, 5, or 6.

All of these models will be further discussed in future empirical studies.

## Results

### Descriptive Statistics

**Figure 3:**
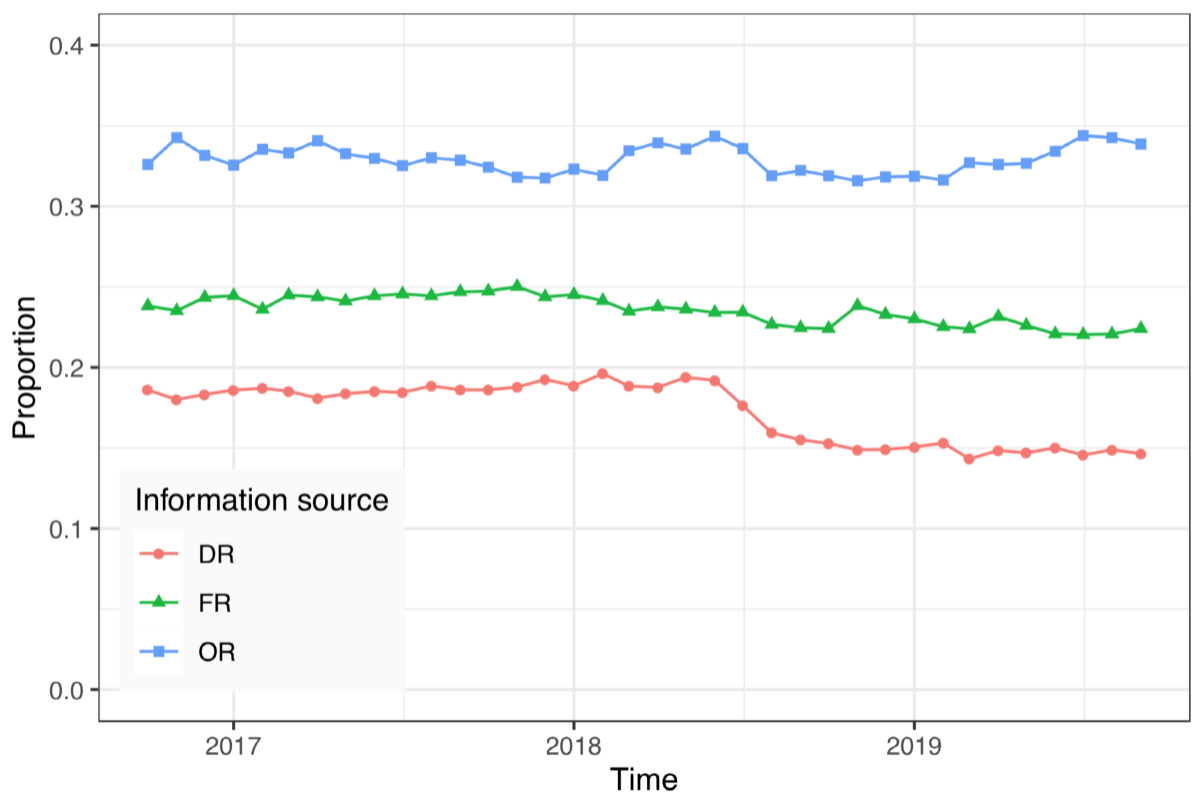
The proportions of the 3 main information sources in each month. DR: doctor recommendations; FR: family and friend recommendations; OR: online reviews.

As shown in Table 1, patients relied primarily on the 3 main information sources, OR, FR, and DR, which accounted for 32.93% (95% CI 32.86%–33.00%; 559,345/1,698,666), 23.68% (95% CI 23.62%-23.75%; 402,322/1,698,666), and 17.48% (95% CI 17.42%-17.54%; 296,912/1,698,666), respectively, when choosing a doctor. To check whether these results were robust, we further divided the sample for the entire 3-year period into 36 subsamples of 1 month. We then similarly examined the proportions of the 3 information sources, OR, FR, and DR, in each subsample. Figure 3 displays the time-varying proportions of these 3 information sources in each month. The results showed proportion values between 31.59% (11,836/37,553) and 34.40% (13,676/39,760) for OR, between 22.04% (8762/39,760) and 25.02% (12,483/49,890) for FR, and between 14.32% (5547/38,723) and 19.61% (8001/40,797) for DR. These results indicate that the proportions of these 3 sources did not change much over time during the sample period.

**Figure 4:**
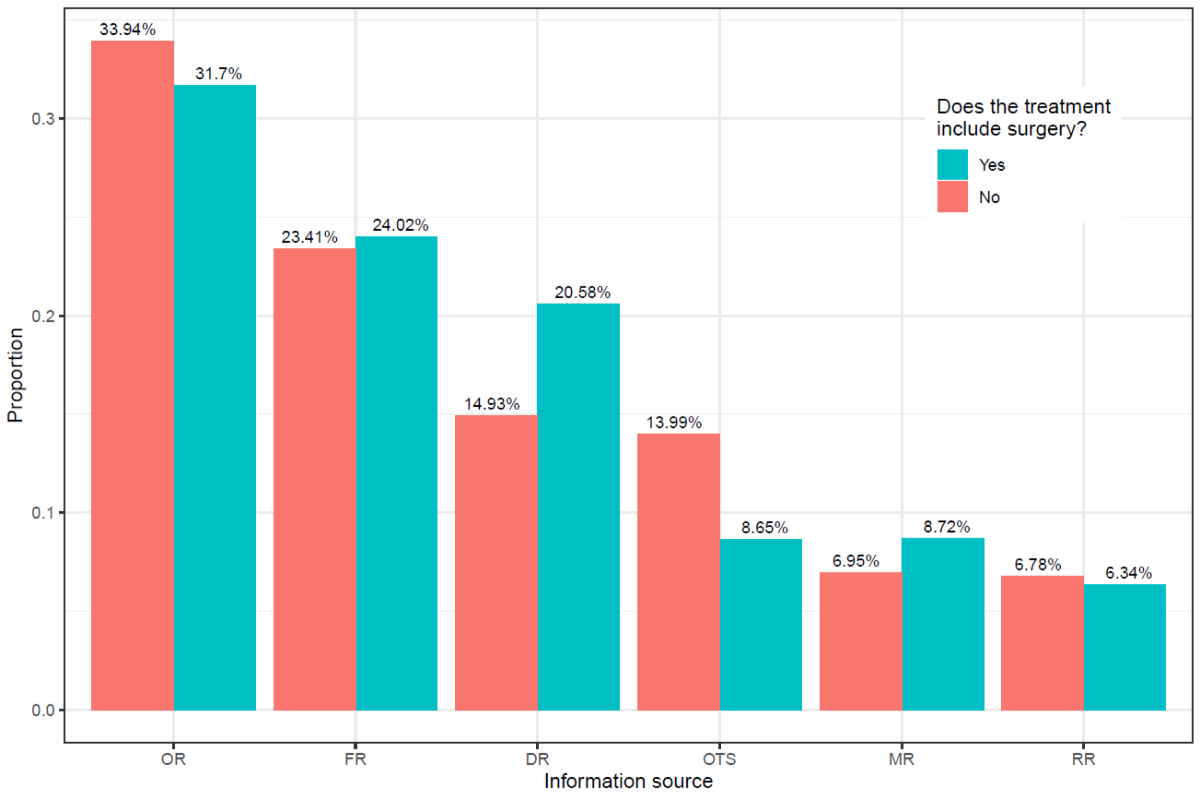
The influence of treatment on reasons for choosing doctors. DR: doctor recommendations; FR: family and friend recommendations; MR: multiple reasons; OR: online reviews; OTS: others; RR: random registration.

**Figure 5:**
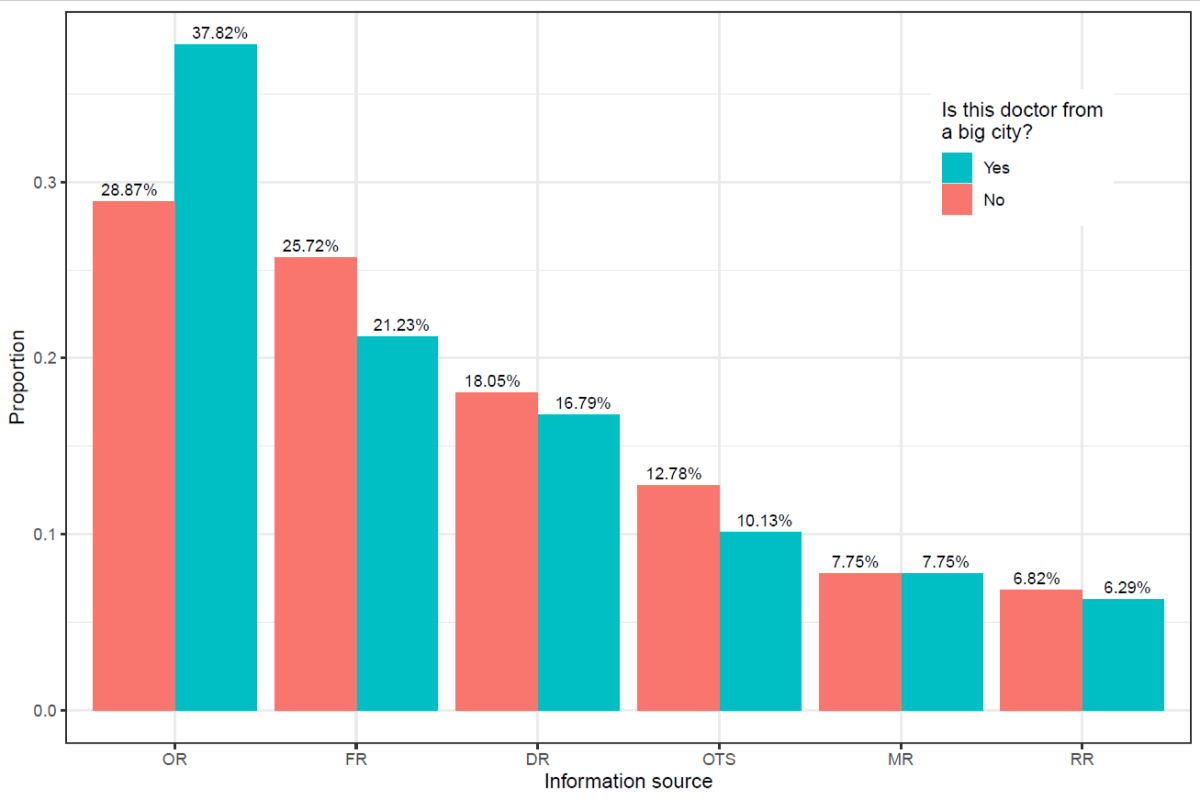
The influence of city on reasons for choosing doctors. DR: doctor recommendations; FR: family and friend recommendations; MR: multiple reasons; OR: online reviews; OTS: others; RR: random registration.

**Figure 6:**
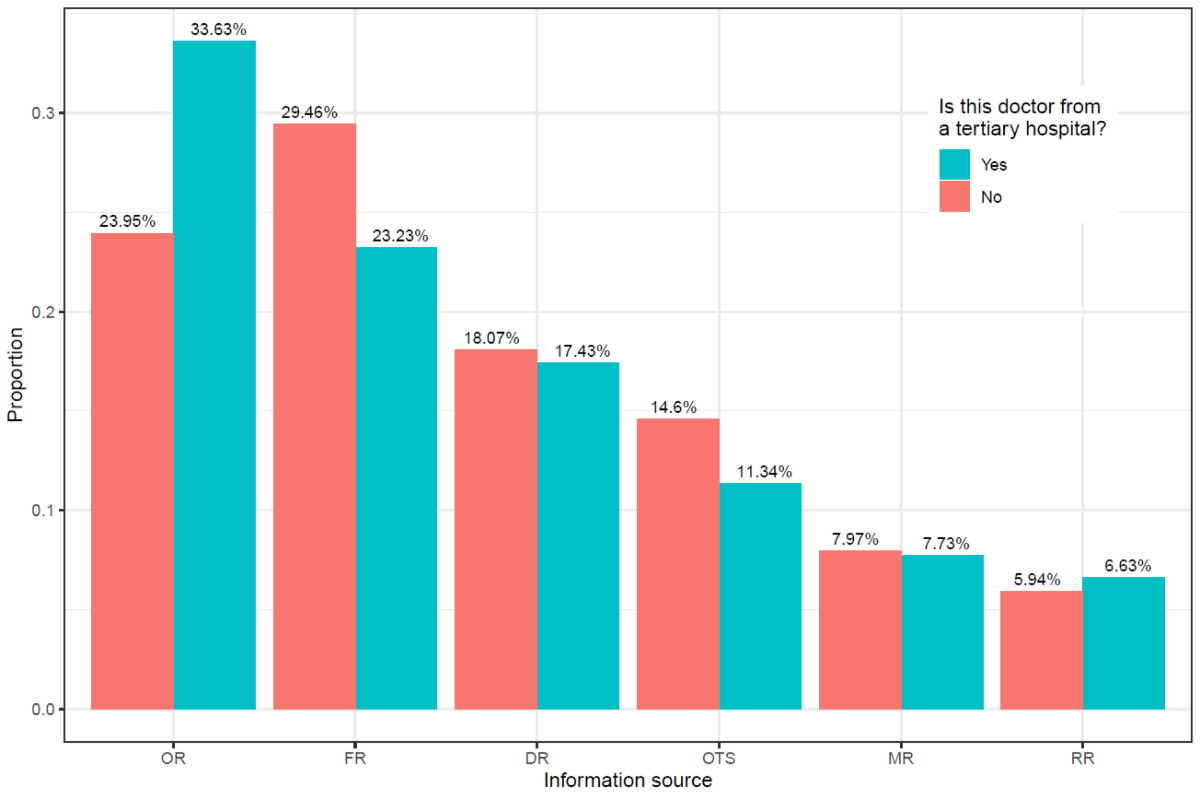
The influence of hospital on reasons for choosing doctors. DR: doctor recommendations; FR: family and friend recommendations; MR: multiple reasons; OR: online reviews; OTS: others; RR: random registration.

In order to clarify the effect of treatment, city, and hospital on proportions in which information sources were used, we first provide visualizations of the results for each of these factors. We take treatment as an example to illustrate how relevant graphics were generated. First, all 1,698,666 samples were divided into 2 subsamples based on treatment, with sample sizes of 766,933 (treatment=1) and 931,733 (treatment=0). We then calculated the weight of different information sources in each subsample (these outcomes are presented in Figure 4). This figure indicates that when patients' treatments included surgery compared with not involving surgery, patients showed greater preference for finding a suitable doctor by using DR (20.58% vs 14.93%; 157,808/766,933 vs 139,104/931,733), FR (24.02% vs 23.41%; 184,293/766,933 vs 218,083/931,733), or MR (8.72% vs 6.95%; 66,859/766,933 vs 64,789/931,733) rather than OR (31.70% vs 33.94%; 243,096/766,933 vs 316,249/931,733), OTS (8.65% vs 13.99%; 66,322/766,933 vs 130,317/931,733), or RR (6.34% vs 6.78%; 48,609/766,933 vs 63,191/931,733). Similarly, Figure 5 shows that when doctors chosen by patients were from a large city compared with not being from a large city, patients showed greater preference for making a decision through OR (37.82% vs 28.87%; 291,332/770,384 vs 268,013/928,282) rather than other sources, especially FR (21.23% vs 25.72%; 163,536/770,384 vs 238,786/928,282). Finally, Figure 6 shows that when doctors chosen by patients were from a tertiary hospital compared with not being from the tertiary hospital, patients showed a strong preference for using OR (33.63% vs 23.95%; 529,779/1,575,216 vs 29,566/123,450) rather than FR (23.23% vs 29.46%; 365,956/1,575,216 vs 36,366/123,450) as their main information source.

While the visual graphs allow us to easily grasp the main findings, we still need to verify the findings’ statistical significance using the multinomial logistic regression model.

### Multinomial Logistic Regressions

Table 2 presents the multinomial logistic regression estimation for model 1, model 2, and model 3 with the 1,698,666 samples. Since the outcome measure in this kind of analysis is the odds ratio, we briefly introduce that concept as follows. An odds ratio greater than 1 indicates that the possibility of the patient using the comparison information source relative to the possibility of the patient using the baseline information source increases as the dummy variable changes from 0 to 1. In other words, the comparison information source is more likely to be used by patients in that case. For example, in model 1, where OR is set as the baseline information source, if we consider FR as the comparison information source, we can observe that the odds ratios of the 3 dummy variables, treatment, city, and hospital, are 1.16, 0.63, and 0.58, respectively. This means that when treatment changes from 0 to 1, patients are more likely to use FR than OR, but when city or hospital changes from 0 to 1, patients are less likely to use FR than OR.

**Table 2:** Multinomial logistic regression results.

Model		Treatment (yes), odds ratio (95% CI)	City (yes), odds ratio (95% CI)	Hospital (yes), odds ratio (95% CI)
**Model 1: Using online reviews as the baseline**				
	Family and friend recommendations	1.16 (1.15-1.17)	0.63 (0.62-0.63)	0.58 (0.57-0.59)
	Doctor recommendations	1.54 (1.53-1.56)	0.69 (0.68-0.69)	0.69 (0.68-0.70)
	Multiple reasons	1.39 (1.37-1.41)	0.74 (0.73-0.75)	0.69 (0.68-0.71)
	Random registration	1.04 (1.03-1.05)	0.70 (0.69-0.71)	0.82 (0.79-0.84)
	Others	0.70 (0.69-0.71)	0.63 (0.63-0.64)	0.59 (0.58-0.60)
**Model 2: Using family and friend recommendations as the baseline**				
	Online reviews	0.86 (0.85-0.87)	1.59 (1.58-1.61)	1.73 (1.71-1.76)
	Doctor recommendations	1.33 (1.31-1.34)	1.09 (1.08-1.10)	1.19 (1.17-1.21)
	Multiple reasons	1.20 (1.18-1.21)	1.18 (1.17-1.20)	1.20 (1.17-1.23)
	Random registration	0.89 (0.88-0.91)	1.12 (1.11-1.14)	1.41 (1.38-1.45)
	Others	0.60 (0.59-0.61)	1.01 (1.00-1.02)	1.02 (1.00-1.04)
**Model 3: Using doctor recommendations as the baseline**				
	Online reviews	0.65 (0.64-0.65)	1.46 (1.45-1.47)	1.45 (1.43-1.48)
	Family and friend recommendations	0.75 (0.75-0.76)	0.92 (0.91-0.93)	0.84 (0.82-0.85)
	Multiple reasons	0.90 (0.89-0.91)	1.08 (1.07-1.10)	1.01 (0.98-1.03)
	Random registration	0.67 (0.66-0.68)	1.03 (1.01-1.04)	1.19 (1.15-1.22)
	Others	0.45 (0.45-0.46)	0.93 (0.91-0.94)	0.85 (0.84-0.87)

Based on the above interpretation, the significance of the odds ratio representations in different situations is clear, so we will not provide additional explanation for each number in Table 2. Nevertheless, there are two points that deserve further elaboration. First, for testing of the statistical significance of each odds ratio, the corresponding 95% confidence interval is also presented in the table. The absence of 1 in the confidence interval for a variable's odds ratio indicates that this variable is significantly associated with the patient's preference for using the comparison or baseline information sources. In Table 2, all odds ratios are significant, except for those in model 3, which has 1 odds ratio that is not significant. Second, we should note that the results in Table 2 are consistent with the findings in Figures 4-6. For example, Figure 4 shows that patients prefer to use OR if the doctor is in a big city. Likewise, in model 1 of Table 2, all odds ratios associated with the city variable are less than 1, meaning that relative to any other information source, patients are more likely to use OR when city changes from 0 to 1. Next, we explore the relationship between different medical specialties and the patients' preference for particular information sources.

### Impacts of Different Medical Specialties

In order to investigate the impact of different medical specialties on patient preferences for using particular information sources to choose doctors, we grouped doctors based on the Chinese names of their departments as listed on the Good Doctor website. Because the total number of categories into which medical specialties can be subdivided is very high, and because even almost identical medical specialties may have differences in Chinese terminology, the number of groups was greater than 2000. While we could try to determine which specialties might be placed in the same category, objectively setting the criteria for such consolidation is a challenge. Hence, we decided not to perform a subjective consolidation process, but instead kept only the 36 medical specialties that had more than 10,000 records for the following analysis. A Chinese and English comparison table of these 36 medical specialty names is available in Multimedia Appendix 1 for readers' reference. This method of categorization can bring several benefits. First, this method does not include any subjective interpretations and retains the full names of medical specialties. It retains complete information about specialties and allows users to explore more possible issues. Second, these medical specialties have a large enough sample size to ensure statistical analytical reliability, and this also shows that these specialties are the most common in China. Third, the current results allow users to build a new category according to their requirements and find the relevant proportions of information sources.

**Figure 7:**
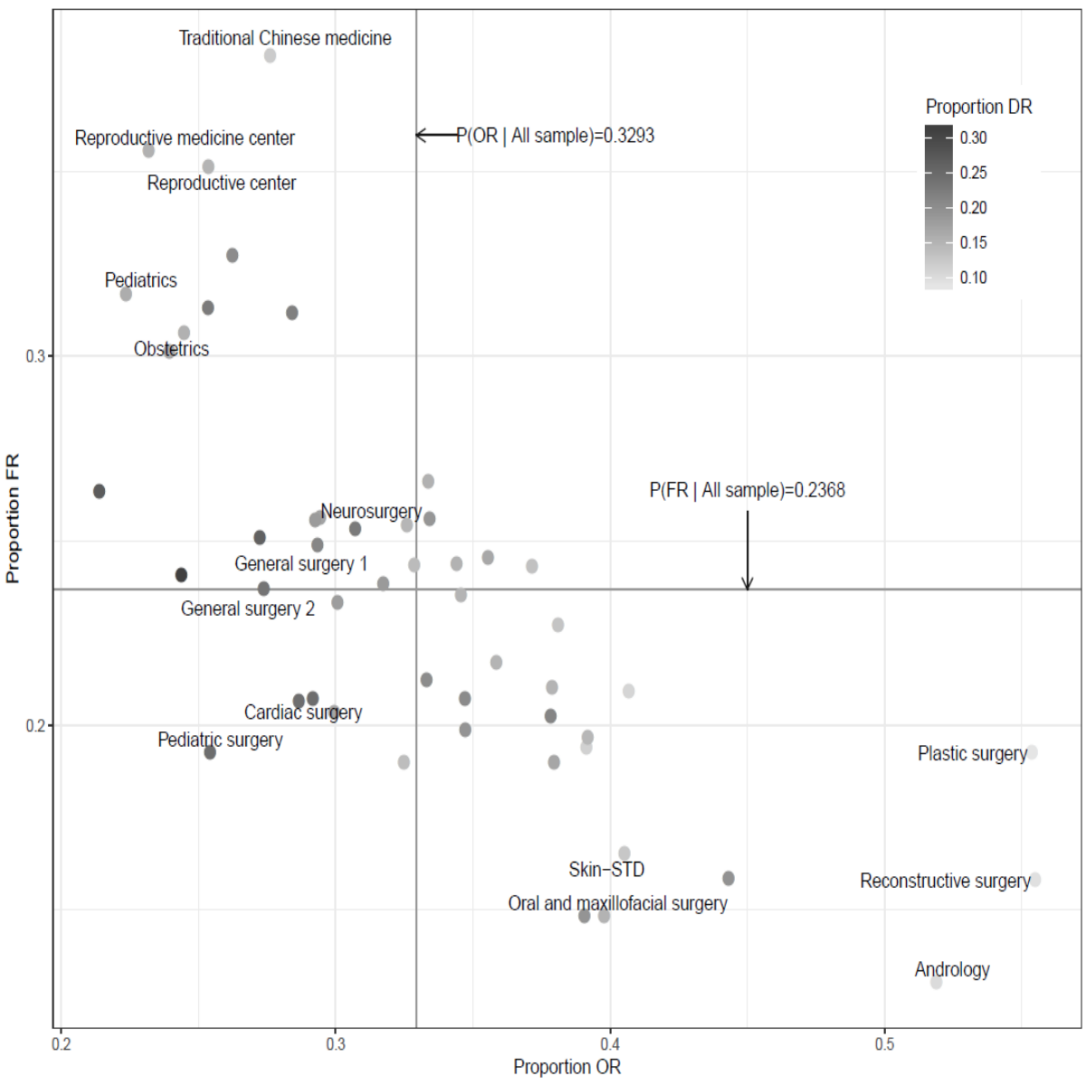
Proportions in the use of information sources for different medical specialties. DR: doctor recommendations; FR: family and friend recommendations; OR: online reviews; STD: sexually transmitted disease.

For example, if we combine neurosurgery and neurology as a new specialty category and measure the proportion of OR for this specialty, according to the outcomes in Table 3, we can easily find that the proportion of OR is 33.45%, which is calculated with (61,199 × 0.3071 + 37,711 × 0.3789) ÷ (61,199 + 37,711). For each medical specialty, we calculated the proportion of each information source and the corresponding 95% confidence interval. The relevant results are presented in Table 3. For example, 90,693 patients saw a urologist, and the proportion of those using OR, FR, DR, MR, OTS, and RR to select a doctor was 34.72% (31,489/90,693), 19.86% (18,012/90,693), 19.14% (17,359/90,693), 8.03% (7283/90,693), 10.07% (9133/90,693), and 8.18% (7419/90,693), respectively. We summarized the proportion of the 36 medical specialties for which the selection of a doctor was made using the 3 most important information sources (ie, OR, FR, and DR) in Figure 7, and we gave the names of the medical specialties with the top 5 highest proportions for selection using each information source. Specifically, in terms of OR, the top 5 medical specialties that showed the highest percentage were reconstructive surgery (9241/16,657, 55.48%), plastic surgery (6209/11,214, 55.37%), andrology (7099/13,680, 51.89%), skin–sexually transmitted disease (7632/18,834, 40.52%), and oral and maxillofacial surgery (5105/12,834, 39.78%). In terms of FR, the order was traditional Chinese medicine (5451/14,282, 38.17%), reproductive medicine center (5048/14,187, 35.58%), reproductive center (4321/12,301, 35.13%), pediatrics (10,370/32,723, 31.69%), and obstetrics (5029/16,425, 30.62%). In terms of DR, the order was pediatric surgery (4818/19,626, 24.55%), cardiac surgery (2742/11,184, 24.52%), general surgery 2 (4043/17,210, 23.49%), neurosurgery (13,197/61,199, 22.74%), and general surgery 1 (6657/31,489, 21.14%). From the above results, it is clear that when patients choose a doctor with a different specialty, they may use different sources of information. A related discussion is included in the next section.

**Table 3:** Comparison of reasons for choosing doctors among various specialties.

Specialty	Observation	OR^a^, % (95% CI)	FR^b^, % (95% CI)	DR^c^, % (95% CI)	MR^d^, % (95% CI)	OTS^e^, % (95% CI)	RR^f^, % (95% CI)
Urology	90,693	34.72 (34.41-35.03)	19.86 (19.60-20.12)	19.14 (18.89-19.40)	8.03 (7.85-8.20)	10.07 (9.87-10.26)	8.18 (8.01-8.36)
Dermatology	77,961	39.13 (38.79-39.47)	19.38 (19.11-19.66)	11.38 (11.15-11.60)	6.68 (6.50-6.85)	14.37 (14.13-14.62)	9.06 (8.86-9.26)
Gynecology	65,551	34.41 (34.04-34.77)	24.35 (24.02-24.68)	15.24 (14.97-15.52)	6.81 (6.62-7.01)	12.83 (12.58-13.09)	6.35 (6.17-6.54)
Orthopedics	64,714	31.73 (31.37-32.09)	23.84 (23.51-24.17)	18.42 (18.13-18.72)	8.38 (8.16-8.59)	10.55 (10.32-10.79)	7.08 (6.88-7.28)
Neurosurgery	61,199	30.71 (30.35-31.08)	25.31 (24.96-25.65)	22.74 (22.41-23.07)	8.84 (8.61-9.06)	7.71 (7.50-7.92)	4.70 (4.53-4.87)
Ophthalmology	51,074	33.31 (32.90-33.72)	21.21 (20.86-21.57)	20.28 (19.93-20.63)	7.90 (7.67-8.13)	10.81 (10.54-11.08)	6.49 (6.28-6.71)
Neurology	37,711	37.89 (37.40-38.38)	21.00 (20.59-21.42)	15.06 (14.70-15.42)	7.05 (6.80-7.31)	12.02 (11.69-12.35)	6.98 (6.72-7.24)
Pediatrics	32,723	22.35 (21.90-22.81)	31.69 (31.18-32.19)	15.99 (15.59-16.39)	7.96 (7.67-8.26)	17.36 (16.95-17.77)	4.64 (4.41-4.87)
General surgery 1	31,489	29.34 (28.84-29.84)	24.86 (24.39-25.34)	21.14 (20.69-21.59)	8.25 (7.95-8.55)	10.13 (9.80-10.46)	6.27 (6.00-6.54)
Obstetrics and gynecology	29,641	23.94 (23.46-24.43)	30.11 (29.59-30.64)	16.39 (15.96-16.81)	7.40 (7.10-7.70)	15.85 (15.43-16.26)	6.31 (6.03-6.59)
Thoracic surgery	29,563	33.43 (32.89-33.96)	25.58 (25.08-26.07)	18.89 (18.44-19.33)	9.10 (8.77-9.42)	7.81 (7.51-8.12)	5.20 (4.95-5.46)
Cardiology	26,792	29.43 (28.88-29.98)	25.62 (25.10-26.14)	17.26 (16.81-17.71)	7.03 (6.73-7.34)	13.52 (13.11-13.92)	7.14 (6.84-7.45)
Otolaryngology	25,257	39.19 (38.59-39.79)	19.67 (19.18-20.16)	14.68 (14.24-15.12)	7.14 (6.82-7.46)	12.76 (12.35-13.17)	6.56 (6.26-6.87)
Endocrinology	24,283	32.86 (32.27-33.45)	24.33 (23.79-24.87)	14.18 (13.74-14.62)	7.24 (6.91-7.57)	13.17 (12.75-13.60)	8.22 (7.87-8.56)
Gastroenterology	23,038	35.85 (35.23-36.47)	21.69 (21.16-22.23)	15.00 (14.54-15.46)	7.09 (6.76-7.42)	13.11 (12.67-13.54)	7.26 (6.92-7.59)
Breast surgery	21,887	32.59 (31.97-33.21)	25.41 (24.84-25.99)	14.83 (14.36-15.30)	7.33 (6.98-7.67)	12.30 (11.86-12.73)	7.54 (7.19-7.89)
Hepatobiliary surgery	20,382	35.55 (34.89-36.20)	24.53 (23.94-25.12)	16.58 (16.07-17.09)	8.48 (8.10-8.86)	8.50 (8.11-8.88)	6.37 (6.03-6.70)
Pediatric surgery	19,626	25.42 (24.81-26.03)	19.23 (18.68-19.79)	24.55 (23.95-25.16)	7.87 (7.49-8.24)	13.55 (13.07-14.03)	9.38 (8.97-9.78)
Skin-STD^g^	18,834	40.52 (39.82-41.22)	16.49 (15.96-17.02)	12.69 (12.21-13.17)	6.95 (6.59-7.31)	15.48 (14.96-15.99)	7.87 (7.49-8.26)
Otorhinolaryngology – head and neck surgery	18,595	37.96 (37.26-38.65)	18.98 (18.41-19.54)	16.85 (16.32-17.39)	7.36 (6.99-7.74)	11.22 (10.77-11.68)	7.63 (7.24-8.01)
Division of rheumatology	17,420	29.25 (28.57-29.92)	25.55 (24.90-26.19)	18.23 (17.66-18.81)	7.63 (7.24-8.03)	13.94 (13.42-14.45)	5.40 (5.07-5.74)
General surgery 2	17,210	27.38 (26.71-28.05)	23.67 (23.04-24.31)	23.49 (22.86-24.13)	8.65 (8.23-9.07)	9.81 (9.36-10.25)	7.00 (6.61-7.38)
Reconstructive surgery	16,657	55.48 (54.73-56.24)	15.78 (15.23-16.34)	9.52 (9.08-9.97)	8.62 (8.19-9.05)	6.79 (6.41-7.17)	3.80 (3.51-4.09)
Obstetrics	16,425	24.47 (23.81-25.13)	30.62 (29.92-31.33)	15.40 (14.85-15.95)	8.10 (7.68-8.51)	17.21 (16.63-17.78)	4.21 (3.90-4.51)
Stomatology	14,541	29.94 (29.20-30.69)	20.32 (19.67-20.98)	16.80 (16.19-17.41)	7.31 (6.89-7.73)	16.68 (16.07-17.28)	8.95 (8.48-9.41)
Spine surgery	14,534	33.38 (32.61-34.14)	26.60 (25.88-27.32)	15.46 (14.87-16.05)	8.72 (8.26-9.18)	8.54 (8.08-8.99)	7.31 (6.88-7.73)
Traditional Chinese medicine	14,282	27.62 (26.88-28.35)	38.17 (37.37-38.96)	12.18 (11.65-12.72)	6.44 (6.04-6.84)	12.39 (11.85-12.93)	3.20 (2.91-3.49)
Reproductive medicine center	14,187	23.18 (22.49-23.88)	35.58 (34.79-36.37)	15.44 (14.84-16.03)	8.26 (7.81-8.71)	12.77 (12.22-13.31)	4.77 (4.42-5.12)
Andrology	13,680	51.89 (51.06-52.73)	13.03 (12.46-13.59)	10.18 (9.67-10.68)	8.93 (8.45-9.40)	10.00 (9.50-10.50)	5.98 (5.58-6.38)
Oral and maxillofacial surgery	12,834	39.78 (38.93-40.62)	14.82 (14.21-15.43)	15.14 (14.52-15.76)	7.52 (7.06-7.98)	11.82 (11.26-12.38)	10.92 (10.38-11.46)
Otolaryngology	12,661	37.15 (36.31-38.00)	24.30 (23.56-25.05)	13.40 (12.81-14.00)	7.94 (7.47-8.41)	10.12 (9.59-10.64)	7.08 (6.64-7.53)
Reproductive center	12,301	25.36 (24.59-26.13)	35.13 (34.28-35.97)	15.04 (14.41-15.67)	7.99 (7.51-8.47)	11.62 (11.05-12.18)	4.86 (4.48-5.24)
Psychiatry	11,980	38.10 (37.23-38.97)	22.71 (21.96-23.46)	13.01 (12.41-13.62)	5.97 (5.54-6.39)	14.14 (13.52-14.76)	6.07 (5.64-6.50)
Plastic surgery	11,214	55.37 (54.45-56.29)	19.23 (18.51-19.96)	8.94 (8.41-9.46)	7.45 (6.97-7.94)	6.40 (5.95-6.86)	2.60 (2.31-2.90)
Cardiac surgery	11,184	29.17 (28.32-30.01)	20.71 (19.96-21.46)	24.52 (23.72-25.31)	8.95 (8.42-9.48)	10.39 (9.82-10.96)	6.27 (5.82-6.72)
Anorectal	10,872	34.57 (33.67-35.46)	23.52 (22.72-24.32)	15.06 (14.38-15.73)	8.31 (7.80-8.83)	11.40 (10.8-11.99)	7.15 (6.66-7.63)

^a^OR: online reviews.

^b^FR: family and friend recommendations.

^c^DR: doctor recommendations.

^d^MR: multiple reasons.

^e^OTS: others.

^f^RR: random registration.

^g^STD: sexually transmitted disease.

## Discussion

### Principal Results

The current study used 1,698,666 samples collected from the Good Doctor Website, including information related to 111,042 doctors, 4747 different hospitals, 1095 observation days, and 31 provinces or municipalities in China; the diversity of these data ensures the empirical results are representative. The main findings can be summarized in three points. First, our results showed that the 3 primary sources of information used by Chinese patients were OR, FR, and DR, accounting for 74.09% (1,258,579/1,698,666) of all usage of sources. Surprisingly, the proportion of use of DR is very low, at only 17.48% (296,912/1,698,666), which may be related to the development of the family doctor service system. In many countries, initial consultations are made through the family doctor service system, followed by referrals to specialists, with the expectation that health resources will be used effectively to control health costs and improve health outcomes [37,38]. Indeed, the Chinese government is also improving the family doctor service system [39,40], but it will not be easy to change the general public's perceptions regarding medical care in a short time. In 2009, China started a new round of health care system reform, in which the development of community health service centers (CHSCs) is a key measure. In the following years, CHSCs had become significantly more functional, but residents did not have sufficient confidence in the primary health care system, and therefore the tiered diagnosis and treatment system was not established as expected. In 2011, the government began to establish a system of family doctors in a number of developed cities based on the existing CHSC service model. Local residents are encouraged to contract with family doctors and use CHSC-based initial consultations for referral services. More information on the Chinese health care system can be found in a recent World Health Organization report [41]. Since our results show that 32.93% (559,345/1,698,666) of patients refer to OR in making their selections, the full use of strategies related to the population's online tendencies may be a direction that health care authorities could consider in promoting the development of the family doctor service system. For example, governments could further liberalize telemedicine regulations to make it easier for people to conduct initial consultations with family doctors over the internet.

Second, understanding the varied preferences of patients in using information sources in different situations can be of aid in the effective dissemination of health care information. For example, if the patient needs surgery, they usually need professional advice or a more trusted information source, such as DR or FR. In other words, when decision making becomes more difficult, people use weak-tie sources like OR less often [32]. Furthermore, as shown in Figure 4, patients are more likely to consider using multiple information sources or to avoid randomly choosing a doctor if they need surgery. In addition, the impact of urban-rural differences is evident. It may be that the information gap leads patients in nonmetropolitan areas to use OR less often than patients in large cities, or it may be that patients in large cities are less likely to have access to strong-tie sources due to more distant relationships and must therefore rely on OR. It is also possible that patients in large cities have a higher level of medical literacy, so they are better able to organize and comprehend information from the internet rather than having to seek advice from family or friends. Finally, we explore the question of whether doctors being from tertiary hospitals has an effect on patient preferences in using information sources. If the physician is not from a tertiary hospital, the patient is more likely to choose a doctor using FR rather than OR. This phenomenon can be explained in terms of brand impact. The effects of a good brand can convince consumers that it features consistent quality, which in turn can reduce consumer perceptions of risk [42]. Tertiary hospitals are like a better brand of the health care system in China. When a doctor is from a tertiary hospital, the patient's risk perception lowers and they are more willing to use online reviews to choose a doctor. Conversely, if the doctor is not from a tertiary hospital, the patient's risk perception rises, and they may need to use more trusted information sources to help them make decisions.

Third, some meaningful outcomes can be found when we look at the 5 medical specialties with the highest weighting in the use of each of the information sources indicated in Figure 7. Related to the use of DR, it is consistent with the above findings that the highest 5 specialties were related to surgery; they include pediatric surgery and cardiac surgery. Related to the use of OR, we find that the medical professions for which patients most prefer to use this information source were associated with personal privacy. Specifically, when patients need the help of this type of medical specialist, they usually gather information about the doctor through the internet and do not want their family or friends to know about it, so specialties with high OR have a low FR. For example, when patients needed the help of an andrologist, 51.89% (7099/13,680) referred to online reviews, and only 13.03% (1783/13,680) sought advice from family or friends. Corresponding to the use of FR, in addition to traditional Chinese medicine, the highest 5 specialties were related to children. It seems likely that friends and family may not necessarily have relevant experience with a particular medical problem, but if they have children, they can certainly share their own medical experience with patients. Another possible reason is that patients tend to be cautious about child-related issues, so they prefer to use strong-tie sources rather than weak-tie sources [32], which also reflects the fact that these specialties related to the high use of FR are also related to low use of OR. As for traditional Chinese medicine, the reason it is related to such a high use of FR is unclear. Perhaps it is because traditional Chinese medicine is very popular in the Chinese world [43], and therefore it is easy to get useful information from friends and family. In addition, in most cases, patients seeking a traditional Chinese medicine doctor usually do not have an urgent medical condition, so they usually have sufficient time to gather information from family or friends to make a choice.

In summary, based on the study's findings, we make 4 specific recommendations for hospital administrators or policymakers in the government health care sector. First, avoiding disruption of the health care system with rumors or untrue advertisements on the internet is an important task because online reviews are currently the main source of information for the general public when they go to the clinic, especially for some medical specialties that are related to personal privacy. Second, the rate of referrals by family doctors still appears to be low, and consideration could be given to the internet habits of the population in conjunction with the family doctor system when the effort is made to change the general public’s medical habits, thereby mitigating overcrowding in hospitals. Third, the gap between urban and rural residents in obtaining health information persists. This may be due to differences in ease of access to medical information or may reflect differences in individuals’ ability to interpret medical knowledge, but people in nonmetropolitan areas make less frequent use of online information when choosing a doctor. The government should continue to strengthen the information infrastructure in rural areas and actively promote health literacy locally. Fourth, people's trust in nontertiary hospitals remains low, so most patients rely primarily on the experience of family and friends when they need to choose a doctor in a nontertiary hospital. The fact that doctors in nontertiary hospitals are also fully capable of treating patients if the disease is not particularly serious is something that administrators must make people aware of.

### Limitations and Future Work

This study has some limitations and points to possible future research questions. First, our sample was drawn from patients or their families who have shared thoughts about their medical visits on the OHC, which means that most of these patients have the ability to use the internet. Thus, our sample selection may have automatically excluded patients who did not have access to the internet, which may have led to the outcomes overestimating the proportion in which patients used online reviews as their information source and underestimating the impact of urban-rural differences. Second, because of ethical considerations, this study did not use any variables related to patients’ personal information. If some personal information about patients can be included in the research model (with prior permission), further insight into the behavioral characteristics involved in the choice of doctor will be gained. Third, because the sample used in this study is entirely from China, differences in culture or social environment may result in the findings of the study not being applicable to other countries or regions. We believe that the difficulty of medical decision making, hospital level, and rural-urban differences are still significantly associated with patients’ preferred information source in most cases. However, the impacts of medical specialties may be significantly different in other countries and regions, a possibility which requires further investigation in the future. Finally, this study examines which information sources patients use to choose their doctors, but it does not attempt to determine how good the decisions are. Specifically, we used a large amount of actual data to explore which information sources patients preferred to use to help them make decisions in different situations. Whether or not those decisions actually led them to the right doctors was a question beyond the scope of our study. Future research could employ textual analysis of the online review content to provide insight into which information sources are used to help patients make better decisions in different contexts.

### Conclusions

Patients’ abilities to make appropriate decisions when seeking a doctor often depend on their own knowledge or characteristic ability to compile relevant information, and the way that they obtain useful information is the first question they must face before a decision can be made. This study investigated the types of information sources that are currently widely used in choosing doctors and showed how the preferred sources vary from situation to situation, thus contributing to an understanding of how to help patients obtain the information they will need in the future. This study makes several specific contributions. First, we know based on a large amount of data that patients currently use online reviews, family and friend recommendations, and doctor recommendations to get the information they need when choosing a doctor. Second, different circumstances correspond to differences in patients’ preferences for information sources. Specifically, when medical decisions become more difficult, as when surgery is required, or when a medical facility is not rated as tertiary, patients are less likely to refer to online evaluations, referring more often in the former case to recommendations given by doctors and in the latter case asking more often for recommendations from family and friends. In addition, rural-urban differences are also associated with differences in patient preferences. Patients in large cities are more likely to use information from online reviews rather than recommendations from family and friends. Third, we explored differences in patient preference for information sources in relation to a variety of medical specialties. For specialties related to personal privacy, online reviews were the most common source of information; for specialties related to children, patients were more likely to refer to the opinions of their family and friends, and for specialties related to surgery, they sought out the advice of doctors more often. Of particular interest is that most traditional Chinese medicine patients chose doctors on the recommendations of their family and friends. These results may not only help the government to further promote the dissemination of medical information but may also aid managers in the health care industry in developing better marketing strategies.

## Appendix

Multimedia Appendix 1 English and Chinese medical specialty name comparison table.

## Abbreviations

CHSC: community health service center
DR: doctor recommendations
FR: family and friend recommendations
MR: multiple reasons
OHC: online health care community
OR: online reviews
OTS: others
RR: random registration
