# Supplementary material for: Exploring Types of Information Sources Used When Choosing Doctors: Observational Study in an Online Health Care Community
Source: J Med Internet Res. 2020 Sep 16;22(9):e20910. doi: 10.2196/20910 (PMC7527935; doi:10.2196/20910)
Supplement: Multimedia Appendix 1 [file jmir_v22i9e20910_app1.docx]

**Multimedia Appendix 1.** English and Chinese medical specialty name comparison table.

| **No** | **Specialty in English** | **Specialty in Chinese** |
| --- | --- | --- |
| 1 | Urology | 泌尿外科 |
| 2 | Dermatology | 皮肤科 |
| 3 | Gynecology | 妇科 |
| 4 | Orthopedics | 骨科 |
| 5 | Neurosurgery | 神经外科 |
| 6 | Ophthalmology | 眼科 |
| 7 | Neurology | 神经内科 |
| 8 | Pediatrics | 儿科 |
| 9 | General Surgery 1 | 普外科 |
| 10 | Obstetrics and Gynecology | 妇产科 |
| 11 | Thoracic Surgery | 胸外科 |
| 12 | Cardiology | 心血管内科 |
| 13 | Otolaryngology | 耳鼻喉科 |
| 14 | Endocrinology | 内分泌科 |
| 15 | Gastroenterology | 消化内科 |
| 16 | Breast Surgery | 乳腺外科 |
| 17 | Hepatobiliary Surgery | 肝胆外科 |
| 18 | Pediatric Surgery | 小儿外科 |
| 19 | Skin-STD | 皮肤性病科 |
| 20 | Otorhinolaryngology-Head and Neck Surgery | 耳鼻咽喉头颈外科 |
| 21 | Division of Rheumatology | 风湿免疫科 |
| 22 | General Surgery 2 | 普通外科 |
| 23 | Reconstructive Surgery | 整复外科 |
| 24 | Obstetrics | 产科 |
| 25 | Stomatology | 口腔科 |
| 26 | Spine Surgery | 脊柱外科 |
| 27 | Traditional Chinese Medicine | 中医科 |
| 28 | Reproductive Medicine Center | 生殖医学中心 |
| 29 | Andrology | 男科 |
| 30 | Oral and Maxillofacial Surgery | 口腔颌面外科 |
| 31 | Otolaryngology | 耳鼻咽喉科 |
| 32 | Reproductive Center | 生殖中心 |
| 33 | Psychiatry | 精神科 |
| 34 | Plastic Surgery | 整形科 |
| 35 | Cardiac Surgery | 心胸外科 |
| 36 | Anorectal | 肛肠科 |
